# Supplementary material for: A three-membered ring approach to carbonyl olefination
Source: Nat Commun. 2017 Oct 23;8:1091. doi: 10.1038/s41467-017-01036-y (PMC5653658; doi:10.1038/s41467-017-01036-y)
Supplement: Supplementary file 1 — Supporting Information [file 41467_2017_1036_MOESM1_ESM.pdf]

Supplementary Methods

General information

Unless otherwise stated, all glassware was flame-dried or oven dried before use and all reactions were performed under an atmosphere of argon. Dichloromethane, acetonitrile, toluene, methanol and DMSO were purchased from Sigma-Aldrich anhydrous grade and used as received; all other solvent were distilled before use. All reagents were used as received from commercial suppliers unless otherwise stated. Reaction progress was monitored by thin layer chromatography (TLC) performed on aluminium plates coated with silica gel F_{254} with 0.2 mm thickness. Visualization was achieved by fluorescence quenching with UV light at 254 nm or by staining using potassium permanganate, phosphomolibdic acid solution, *p*-anisaldehyde solution or vanillin solution and heating. Flash column chromatography was performed using silica gel 60 (230-400 mesh, Merck and co.). All ^{1}H NMR and ^{13}C NMR spectra were recorded using a Bruker AV-400 or AV-600 spectrometer at 300K. Chemical shifts were given in parts per million (ppm, δ), referenced to the solvent peak of $CDCl_3$, defined at δ = 7.26 ppm (^{1}H NMR) and δ = 77.16 (^{13}C NMR). Coupling constants are quoted in Hz (*J*). ^{1}H NMR splitting patterns were designated as singlet (s), doublet (d), triplet (t), quartet (q), pentet (p). Splitting patterns that could not be interpreted or easily visualized were designated as multiplet (m) or broad (br). IR spectra were recorded neat on a Perkin-Elmer Spectrum 100 FT-IR spectrometer.

Triethylsulfoxonium chloride and triisopropylsulfoxonium tetrafluoroborate were prepared as described in literature[1-2].

Hydrazones **1c** and **1d** were prepared as described in literature[3-4].

All of the diasteromeric ratios provided in the main text are determined on crude mixtures by NMR integrations of the olefin protons or the allylic protons where not possible.

The yields provided refer to isolated and analytically pure materials, calculated from the initial aldehyde over two or three synthetic steps.

Azines of general structure 2 were never isolated throughout the present work but their formation and quantification is possible on crude mixture thanks to their typical aldimine-like proton (in the range 7.8-8.1 ppm).

Attempts at the synthesis of compounds **6a**, **6b** and **6c** were made using the standard methodologies. Method A was used for **6a** and **6b**, with triisopropylsulfoxonium

tetrafluoroborate and trimehtylsulfoxonium iodide respectively. Method B was used instead for **6c** with trimethylsulfoxonium iodide. The formation of the azine involved in the synthesis of **6a** was inferred from the crude spectra of the reaction mixture and not isolated, as usual in the present work. Compounds **SI-1** involved in the synthesis of **6b** and **SI-2** involved in the synthesis of **6c** were isolated instead, to ensure the structure and the complete characterization is provided below.

Experimental Procedures

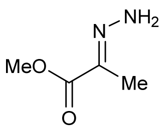

methyl-2-hydrazonopropanoate (1a):

To hydrazine hydrate (2.0 equiv.) was added dropwise a mixture of acetic acid (0.5 M) and water (0.5 M) at 0 °C. The α-keto ester (1.0 equiv.) was added to the mixture at room temperature. The reaction mixture was stirred at room temperature until completion of the reaction as judged by TLC. The volatiles were removed under reduced pressure. Deionized water was added to the residue and the mixture was extracted with ethyl acetate. The combined organic phases were washed with a saturated aqueous solution of sodium hydrogen carbonate, brine and dried over $MgSO_4$. The solvent was removed under reduced pressure to give off white solid product (60% yield) that can be used in the next step. Data in accordance with the literature.[5]

^{1}H-NMR (400 MHz, CDCl3): δ 5.95 (2H, bs, H_5), 3.76 (3H, s, H_7), 2.19 (3 H, s, H_3).

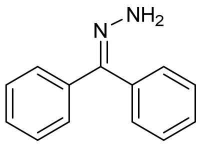

(diphenylmethylene)hydrazone (1b):

A mixture of benzophenone (1.0 equiv.), hydrazine hydrate (2.0 equiv.) and EtOH (0.5 M) was refluxed overnight. The mixture was left to cool to room temperature over 1 h. The solvent was removed under reduce pressure. The obtained solid were recrystallized from absolute EtOH or purified by to column chromatography if necessary. Data in accordance with the literature.[6]

^{1}H-NMR (400 MHz, $CDCl_3$): δ 7.52-7.38 (m, 10H), 5.42 (bs, 2H).

General procedure for olefin synthesis A

A mixture of aldehyde (1.0 equiv.) and hydrazone **1a** (1.1 equiv.) was stirred in the presence of $MgSO_4$ (100 mg per mmol) in MeOH (0.3 M) at reflux per 16 h. Then, the reaction mixture was filtered and the solvent was removed under reduced pressure. In another flask potassium *tert*-butoxide (2.0 equiv.) was added to a solution of trimethyl sulfoxonium iodide (2.5 equiv.) in MeCN (0.2 M). The resulting mixture was stirred for 30 minutes at room temperature. To this solution was added the previously formed azine in MeCN (0.2 M) and the reaction mixture stirred at room temperature until complete conversion. Toluene (0.2 M) was then added and the reaction mixture was heated to 90 °C and stirred at this temperature 16 h. The reaction was then diluted with a saturated aqueous solution of NH_4Cl and extracted with EtOAc (3x). The combined organic layers were washed with brine, dried over anhydrous Na_2SO_4 and concentrated under reduced pressure. The crude product was purified by flash column chromatography on silica gel to afford the desired product.

General procedure for olefin synthesis B

A mixture of aldehyde (1.0 equiv.) and hydrazone **1b** (1.1 equiv.) was stirred in the presence of $MgSO_4$ (100 mg per mmol) in CH_2Cl_2 (0.3 M) at room temperature for 30 min. Then, the reaction mixture was filtered and the solvent was removed under reduced pressure. In pressure vial, potassium *tert*-butoxide (1.5 equiv.) was added to a solution of trimethyl sulfoxonium iodide (1.75 equiv.) in MeCN (0.3 M). The resulting mixture was stirred for 30 minutes at room temperature. To this solution was added the previously formed azine in MeCN (1 M) and the reaction mixture stirred at room temperature until complete conversion. At completion of the reaction the volatiles were removed under reduced pressure and the solid residues were diluted with toluene (0.5 M) and heated to 150 °C. After 24 h the reaction is generally complete and the mixture is cooled to room temperature. The reaction was then diluted with a saturated aqueous solution of NH_4Cl and extracted with EtOAc (3x). The combined organic layers were washed with brine, dried over anhydrous $MgSO_4$ and concentrated under reduced pressure. The crude product was purified by flash column chromatography on silica gel to afford the desired product.

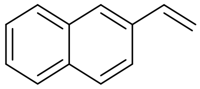

2-vinylnaphthalene (3a):

The title compound was prepared using general procedure A in 84% yield. Data in accordance with the literature.[7]

^{1}H-NMR (400 MHz, $CDCl_3$) δ 7.79-7.74 (m, 3H), 7.76 (s, 1H), 7.65 (dd, *J* = 8.8 1.7 Hz, 1H), 7.50-7.42 (m, 2H), 6.90 (ddd, *J* = 17.6, 10.9, 0.5 Hz, 1H), 5.89 (dd, *J* = 17.6, 0.7 Hz, 1H), 5.35 (dd, *J* = 10.9, 0.9 Hz, 1H).

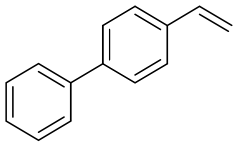

4-vinyl-1,1'-biphenyl (3b):

The title compound was prepared using general procedure A in 88% yield. Data in accordance with the literature.[7]

^{1}H-NMR (400 MHz, $CDCl_3$) δ 7.60-7.56 (m, 4H), 7.50-7.42 (m, 4H), 7.37-7.33 (m, 1H), 6.77 (dd, *J* =17.6, 10.5 Hz, 1H), 5.80 (dd, *J* =17.6, 0.9 Hz, 1H), 5.28 (dd, *J* = 10.5, 0.9 Hz, 1H).

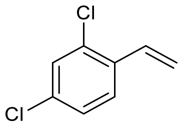

2,4-dichloro-1-vinylbenzene (3c):

The title compound was prepared using general procedure A in 80% yield. Data in accordance with the literature.[8]

^{1}**H-NMR (400 MHz, $CDCl_3$)** δ 7.49 (d, *J* = 8.5 Hz, 1H), 7.38 (d, *J* = 2.1 Hz, 1H), 7.21 (dd, *J* = 8.5, 2.1 Hz, 1H), 7.03 (dd, *J* = 17.6, 11.1 Hz, 1H), 5.73 (d, *J* = 17.2 Hz, 1H), 5.40 (d, *J* = 11.1 Hz, 1H).

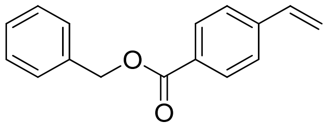

benzyl 4-vinylbenzoate (3d):

The title compound was prepared using general procedure A in 72% yield. Data in accordance with the literature. [9]

^{1}**H-NMR (400 MHz, $CDCl_3$)** δ 8.03 (d, *J*= 8.5 Hz, 2H), 7.47-7.44 (m, 3H), 7.41-7.33 (m, 4H), 6.75 (dd, *J* = 17.5, 10.9 Hz, 1H), 5.86 (d, *J* = 17.5 Hz, 1H), 5.37 (d, *J* = 10.9 Hz, 1H) 5.36 (s, 2H).

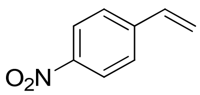

1-nitro-4-vinylbenzene (3e):

The title compound was prepared using general procedure A in 30% yield. Data in accordance with the literature.[10]

^{1}**H-NMR (400 MHz, $CDCl_3$)** δ 8.19 (d, *J* = 9.3 Hz, 2H), 7.55 (d, *J* = 8.8 Hz, 2H), 6.78 (dd, *J* = 18.2, 11.0 Hz, 1H), 5.93 (d, *J* = 17.9 Hz, 1H), 5.50 (d, *J* = 10.9 Hz, 1H).

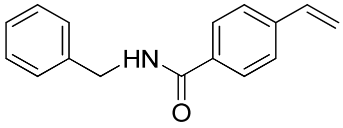

N-benzyl-4-vinylbenzamide (3f):

The title compound was prepared using general procedure A in 81% yield. Data in accordance with the literature.[11]

^{1}H-NMR (400 MHz, $CDCl_3$) δ 7.75 (d, *J* = 8.2 Hz, 2H), 7.45 (d, *J* = 8.2 Hz, 1H), 7.36 (m, 4H), 7.34-7.22 (m, 1H) 6.74 (dd, *J* = 17.5, 10.8 Hz, 1H), 6.37 (bs, 1H), 5.83 (d, *J* = 17.5 Hz, 1H), 5.35 (d, *J* = 10.8 Hz, 1H), 4.65 (d, *J* = 5.6 Hz, 2H).

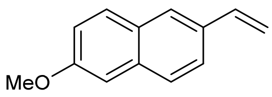

2-methoxy-6-vinylnaphthalene (3g):

The title compound was prepared using general procedure A in 80% yield. Data in accordance with the literature.[7]

^{1}H-NMR (400 MHz, $CDCl_3$) δ 7.72-7.69 (m, 3H), 7.60 (dd, *J* = 8.7, 1.5 Hz, 1H), 7.15-7.12 (m, 2H), 6.85 (dd, *J* = 17.8, 10.8 Hz, 1H), 5.82 (dd, *J* = 17.6, 0.7 Hz, 1H), 5.28 (dd, *J* = 10.5, 0.7 Hz, 1H, $H_{13'}$) 3.92 (s, 3H).

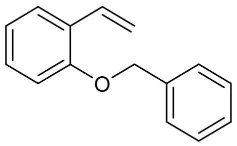

1-(benzyloxy)-2-vinylbenzene (3h):

The title compound was prepared using general procedure A in 79% yield. Data in accordance with the literature.[12]

^{1}H-NMR (400 MHz, $CDCl_3$) δ 7.51 (dd, *J* =7.6, 1.7 Hz, 1H), 7.47-7.44 (m, 2H), 7.42-7.37 (m, 2H), 7.35-7.30 (m, 1H), 7.22 (td, *J* = 8.1, 1.7 Hz, 1H), 7.14 (dd, *J* = 17.8, 11.3 Hz, 1H), 6.98-6.93 (m, 2H) 5.76 (dd, *J* = 17.7, 1.5 Hz, 1H), 5.26 (dd, *J* = 17.7, 1.5 Hz, 1H), 5.11 (s, 2H).

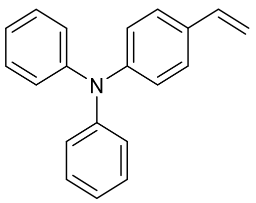

***N*,*N*-diphenyl-4-vinylaniline (3i):**

The title compound was prepared using general procedure A in 74% yield. Data in accordance with the literature.[13]

^{1}H-NMR (400 MHz, $CDCl_3$) δ 7.30-7.352 (m, 6H), 7.10-7.08 (m, 4H), 7.03-7.00 (m, 4H), 6.67 (dd, J = 17.4, 10.7 Hz, 1H), 5.64 (d, J = 17.4 Hz, 1H), 5.16 (d, J = 10.7 Hz, 1H).

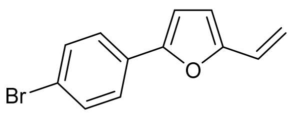

2-(4-bromophenyl)-4-vinylfuran (3j):

The title compound was prepared using general procedure A in 86% yield, as bright yellow crystals**.**

R_f = 0.85 (heptane/EtOAc 95:5, stain with $KMnO_4$);

^{1}H-NMR (400 MHz, $CDCl_3$) δ 7.57-7.49 (m, 4H), 6.64 (d, J = 3.4 Hz, 1H), 6.52 (dd, J = 17.5, 11.3 Hz, 1H), 6.34 (d, J = 3.4 Hz, 1H), 5.76 (dd, J = 17.5, 0.8 Hz, 1H), 5.22 (dd, J = 11.3, 0.8 Hz, 1H);

^{13}C-NMR (101 MHz, $CDCl_3$) δ 153.19, 152.37, 131.97, 129.75, 125.43, 124.98, 121.31, 112.77, 110.53, 107.50;

IR (neat) ν max cm^{-1}:3100, 3054, 2924, 2854, 1676, 1638, 1542, 1522, 1475, 1404, 1281, 1198, 1073, 1008, 824, 783.

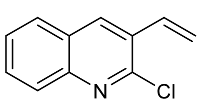

2-chloro-3-vinylquinoline (3k):

The title compound was prepared using general procedure A in 75% yield. Data in accordance with the literature.[14]

^{1}H-NMR (400 MHz, $CDCl_3$) δ 8.20 (s, 1H), 7.92 (d, *J* = 8.5 Hz, 1H), 7.75 (d, *J* = 8.5 Hz, 1H), 7.63 (td, *J* = 7.5, 1.1 Hz, 1H), 7.48 (td, *J* = 7.5, 1.1 Hz, 1H),7.16 (dd, *J* = 17.3, 10.9 Hz, 1H), 5.81 (dd, *J* = 17.3, 0.9 Hz, 1H), 5.47 (dd, *J* = 10.9, 0.9 Hz, 1H).

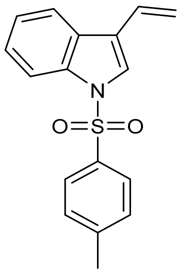

1-tosyl-3-vinyl-1H-indole (3l):

The title compound was prepared using general procedure A in 89% yield. Data in accordance with the literature.[15]

^{1}H-NMR (400 MHz, $CDCl_3$) δ 8.01-7.97 (m, 1H), 7.79-7.72 (m, 3H), 7.60 (s, 1H), 7.36-7.30 (m, 1H), 7.30-7.24 (m, 1H), 7.22 (dd, *J* = 8.6, 0.6 Hz, 2H), 6.77 (ddd, *J* = 17.8, 11.3, 0.7 Hz, 1H), 5.79 (dd, *J* = 17.8, 0.9 Hz, 1H), 5.35 (dd, *J* = 11.3, 1.1 Hz, 1H), 2.34 (s, 3H).

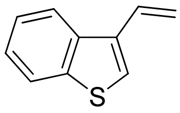

3-vinylbenzo[*b*]thiophene (3m):

The title coumpound was prepared using general procedure A in 85% yield. Data in accordance with the literature.[16]

^{1}H-NMR (400 MHz, $CDCl_3$) δ 7.92 (d, *J* = 8.5 Hz, 1H), 7.87 (d, *J* = 7.8 Hz, 1H) 7.47 (s, 1H), 7.44-7.35 (m, 2H), 6.99 (ddd, *J* = 17.6, 11.1, 0.7 Hz, 1H), 5.82 (dd, *J* = 17.6, 1.3 Hz, 1H), 5.35 (dd, *J* = 11.1, 1.3 Hz, 1H).

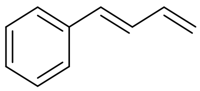

(*E*)-buta-1,3-dien-1-ylbenzene (4a):

The title compound was prepared using general procedure A in 60% yield. Data in accordance with the literature.[17]

^{1}H NMR (400 MHz, $CDCl_3$) δ 7.42-7.21 (m, 5H), 6.84-6.74 (m, 1H), 6.60-6.45 (m, 2H), 5.34 (dd, *J* = 0.9, 16.8 Hz, 1H), 5.17 (d, *J* = 10.2 Hz, 1H).

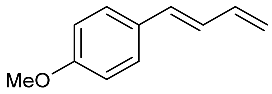

(*E*)-1-(buta-1,3-dien-1-yl)-4-methoxybenzene (4b):

The title compound was prepared using general procedure A in 45% yield. Data in accordance with the literature.[18]

^{1}H NMR(400 MHz, $CDCl_3$) δ 7.34 (d, 2H, *J* = 8.4 Hz), 6.85 (d, *J* = 8.1 Hz, 2H), 6.67 (dd, *J* = 15.0, 10.8 Hz, 1H), 6.51 (d, *J* = 15.0 Hz, 1H), 6.42-6.35 (m, 1H), 5.27 (d, *J* = 16.2 Hz, 1H), 5.11 (d, *J* = 9.9 Hz, 1H), 3.80 (s, 3H).

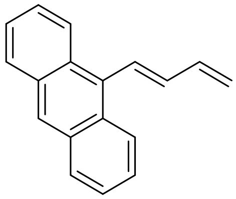

(*E*)-9-(buta-1,3-dien-1-yl)anthracene (4c):

The title compound was prepared using general procedure A in 65% yield. Data in accordance with the literature.[19]

^{1}H NMR: (400 MHz, $CDCl_3$) δ 8.40 (s, 1H), 8.34-8.26 (m, 2H), 8.07-7.98 (m, 2H), 7.56-7.46 (m, 4H), 7.41 (d, *J* = 16.1 Hz, 1H), 6.84 (td, *J* = 16.9, 10.3 Hz, 1H), 6.65 (dd, *J* = 16.0, 10.3 Hz, 1H), 5.40 (dd, *J* = 16.0, 0.7 Hz, 1H), 5.34 (d, *J* = 10.3 Hz, 1H).

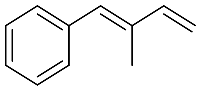

(*E*)-(2-methylbuta-1,3-dien-1-yl)benzene (4d):

The title compound was prepared using general procedure A in 50% yield. Data in accordance with the literature.[20]

^{1}H NMR: (400 MHz, $CDCl_3$) δ 7.38-7.21 (m, 5H,), 6.57 (ddt, *J* = 17.3, 10.8, 1.0 Hz, 1H), 6.55 (s, 1H), 5.32 (d, *J* = 17.3 Hz, 1H), 5.15 (d, *J* = 10.8 Hz, 1H), 2.02 (d, *J* = 1.1 Hz, 3H).

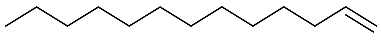

Tridec-1-ene (5a):

The title compound was prepared using general procedure B in 66% yield. Data in accordance with the literature.[21]

^{1}H NMR (300 MHz, $CDCl_3$) δ 5.8-5.7 (m, 1H), 5.0-4.8 (m, 2H), 2.1-2.0 (m, 2H), 1.3–1.1 (m, 18H), 0.8 (t, *J* = 6.7 Hz, 3H).

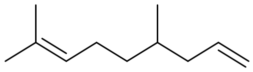

4,8-Dimethylnona-1,7-diene (5b):

The title compound was prepared using general procedure B in 62% yield. Data in accordance with the literature.[22]

^{1}H-NMR (400 MHz, $CDCl_3$) δ 5.79 (ddt, *J* = 17.2, 10.3, 7.1 Hz, 1H), 5.14-5.09 (m, 1H), 5.03-4.99 (m, 1H), 4.99-4.97 (m, 1H), 2.12-1.87 (m, 4H), 1.70 (s, 3H), 1.62 (s, 3H), 1.56-1.48 (m, 1H), 1.41-1.32 (m, 1H), 1.21-1.12 (m, 1H), 0.90 (d, *J* = 6.7 Hz, 3H).

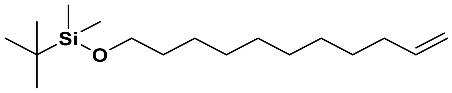

***tert*-Butyldimethyl(undec-10-en-1-yloxy)silane (5c):**

The title compound was prepared using general procedure B in 67% yield. Data in accordance with the literature.[23]

^{1}H-NMR (600 MHz, $CDCl_3$) δ 5.81 (ddt, *J* = 16.9, 10.2, 6.7 Hz, 1H), 5.01-4.91 (m, 2H), 3.59 (t, *J* = 6.7 Hz, 2H), 2.06-2.02 (m, 2H), 1.53-1.48 (m, 2H), 1.38-1.35 (m, 2H), 1.28 (bs, 10H), 2.89 (s, 9H), 0.05 (s, 6H).

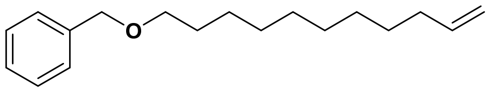

((Undec-10-en-1-yloxy)methyl)benzene (5d):

The title compound was prepared using general procedure B in 57% yield. Data in accordance with the literature.[24]

^{1}H-NMR (400 MHz, $CDCl_3$) δ 7.34-7.21 (m, 5H), 5.81 (ddt, *J* = 16.9, 10.2, 6.7 Hz, 1H), 5.01-4.92 (m, 2H), 4.50 (s, 2H), 3.46 (t, *J* = 6.7 Hz, 2H), 2.06-2.01 (m, 2H), 1.65-1.58 (m, 2H), 1.32-1.39 (m, 2H), 1.28 (bs, 10H).

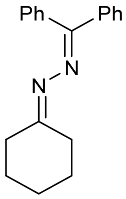

1-Cyclohexylidene-2-(diphenylmethylene)hydrazine (SI-1):

^{1}H-NMR (400 MHz, $CDCl_3$) δ 7.69-7.66 (m, 2H), 7.43-7.34 (m, 6H), 7.24-7.21 (m, 2H), 2.51 (bt, *J* = 6.15 Hz, 2H), 2.25 (bt, *J* = 6.15 Hz, 2H), 1.69-1.61 (m, 6H).

^{13}C-NMR (101 MHz, $CDCl_3$) δ 165.00, 159.28, 138.46, 135.50, 129.59, 129.27 (2C), 128.61, 128.51 (2C), 128.17 (2C), 127.89 (2C), 35.44, 29.15, 27.29, 26.24, 25.87.

IR (neat) ν max cm^{-1}: 3058, 3026, 2929, 2856, 1618, 1560, 1491, 1444, 1318, 1245, 1074.

HRMS (ESI) calcd. for $C_{14}H_{15}O$ $[M+H]^+$: 277.1699, found: 277.1692.

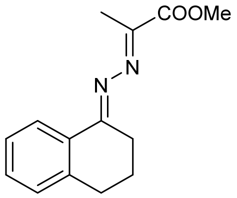

Methyl (*E*)-2-(((*E*)-3,4-dihydronaphthalen-1(2*H*)-ylidene)hydrazono)propanoate (SI-2):

^{1}H-NMR (400 MHz, $CDCl_3$) δ 8.21 (dd, *J* = 7.8, 1.2 Hz, 1H), 7.28 (m, 2H), 7.17 (dd, *J* = 7.5, 0.7 Hz, 1H), 3.90 (s, 3H), 2.82 (t, *J* = 6.1 Hz, 2H), 2.57 (bt, *J* = 6.5 Hz, 2H), 2.08 (s, 3H), 1.94-1.88 (m, 2H).

^{13}C-NMR (101 MHz, $CDCl_3$) δ165.07, 155.36, 149.71, 140.97, 131.92, 130.23, 128.86, 126.57, 126.04, 52.87, 29.78, 27.77, 22.04, 14.25.

IR (neat) ν max cm^{-1}: 3054, 2951, 1719, 1612, 1584, 1482, 1438, 1316, 1265, 1195, 1083.

HRMS (ESI) calcd. for $C_{14}H_{15}O$ $[M+H]^+$: 245.3015, found: 245.1290.

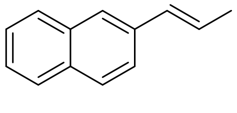

(*E*)-2-(Prop-1-en-1-yl)naphthalene (7a):

The title compound was prepared using general procedure A in 80% yield using triethylsulfoxonium iodide. Data in accordance with the literature.[25]

^{1}H-NMR (400 MHz, $CDCl_3$) δ 7.77 (t, *J* = 8.9 Hz, 2H), 7.67 (s, 1H), 7.57 (d, *J* = 8.5 Hz, 1H), 7.46-7.29 (m, 3H), 6.57 (d, *J* = 15.6 Hz, 1H), 6.42-6.33 (m, 1H), 2.05 (d, *J* = 6.6 Hz, 3H);

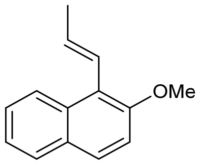

(*E*)-2-Methoxy-1-(prop-1-en-1-yl)naphthalene (7b):

The title compound was prepared using general procedure A in 71% yield using triethylsulfoxonium iodide.

^{1}H-NMR (400 MHz, $CDCl_3$) δ 8.17 (bd, *J* = 8.5 Hz, 1H), 7.77 (bd, *J* = 8.0 Hz, 1H), 7.74 (d, *J* = 9.0 Hz, 1H), 7.44 (ddd, *J* = 8.5, 6.8, 1.4 Hz, 1H), 7.33 (ddd, *J* = 8.0, 6.8, 1.2 Hz, 1H), 7.27 (d, *J* = 9.0 Hz, 1H), 6.76 (dq, *J* = 16.0, 1.8 Hz, 1H), 6.20 (dq, *J* = 16.0, 6.6 Hz, 1H), 3.95 (s, 3H), 2.05 (dd, *J* = 6.6, 1.8 Hz, 3H).

^{13}C-NMR (101 MHz, $CDCl_3$) δ 154.16, 132.85, 132.34, 129.47, 128.31, 128.23, 126.27, 124.70, 123.93, 123.51, 121.63, 113.51, 56.65, 19.60.

IR (neat) ν max cm^{-1}: 2999, .2929, 2852, 1733, 1620, 1591, 1510, 1463, 1376, 1261, 1249, 1087, 967, 806, 746.

HRMS (ESI) calcd. for $C_{14}H_{15}O$ $[M+H]^+$: 199.1117, found: 299.1116.

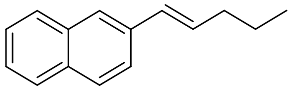

(*E*)-2-(Pent-1-en-1-yl)naphthalene (7c):

The title compound was prepared using general procedure A in 71% yield using tributylsulfoxonium iodide. Data in accordance with the literature.[26]

^{1}H-NMR (400 MHz, $CDCl_3$) δ 7.83-7.76 (m, 3H), 7.68 (s, 1H), 7.59 (dd, *J* = 8.5, 1.7 Hz, 1H), 7.48-7.40 (m, 2H), 6.60-6.54 (m, 1H), 2.29-2.23 (m, 2H), 1.55 (m, 2H), 1.00 (t, 7.0 Hz, 3H).

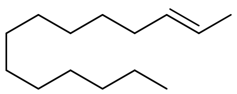

(*E*)-Tetradec-2-ene (7d):

The title compound was prepared using general procedure A in 71% yield using triethylsulfoxonium iodide. Data in accordance with the literature.[27]

^{1}H-NMR (400 MHz, $CDCl_3$) δ 5.54-5.25 (m, 2H), 2.03 (dd, *J* = 12.7, 6.2 Hz, 2H), 1.61 (m, 3H), 1.27 (bs, 18H) 0.89 (t, *J* = 6.9 Hz, 3H).

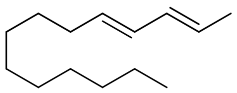

(2*E*,4*E*)-Tetradeca-2,4-diene (7e):

The title compound was prepared using general procedure A in 71% yield using triethylsulfoxonium iodide. Data in accordance with the literature.[28]

^{1}H-NMR (400 MHz, $CDCl_3$) δ 6.05-6.03 (bd, 2H), 5.88 – 5.78 (m, 1H), 5.59 – 5.51 (m, 1H), 2.20-2.15 (m, 2H), 1.81 (m, 3H), 1.30-1.23 (bm, 14H). 0.88 (t, *J* = 6.7 Hz, 3H).

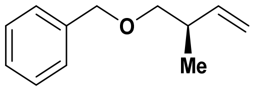

(*R*)-(((2-Methylbut-3-en-1-yl)oxy)methyl)benzene (8):

The title compound was prepared using general procedure B in 60% yield. Data in accordance with the literature.[29]

^{1}H-NMR (600 MHz, $CDCl_3$) δ 7.35-7.27 (m, 5H), 5.81 (ddd, *J* = 17.3, 10.4, 6.9 Hz, 1H), 5.10-5.00 (m, 2H), 4.52 (s, 2H), 3.35 (ddd, *J* = 31.4, 9.1, 6.7 Hz, 2H), 2.56-2.47 (m, 1H), 1.05 (d, *J* = 6.8 Hz, 3H).

Enantiomeric ratio 98.9:1.1 was determined by chiral HPLC analysis, Chiralpak IB 250x4.6 mm ID, *n*-hexane + 0.1% *i*-PrOH, 0.5 ml/min, 20 °C, detection at 210 nm, retention times (min): 11.56 (major), 12.19 (minor).

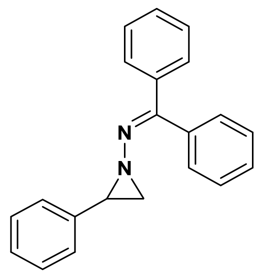

1,1-Diphenyl-*N*-(2-phenylaziridin-1-yl)methanimine (9):

A mixture of benzalaldehyde (108 mg, 1.02 mmol, 1.0 equiv.) and hydrazone **1b** (200 mg, 1.02, 1.0 equiv.) was stirred in the presence of $MgSO_4$ in CH_2Cl_2 at room temperature for 1h. Then, the reaction mixture was filtered, the solvent was removed under reduced pressure. In another flask Potassium *tert*-butoxide (229 mg, 2.04 mmol, 2.0 equiv.) was added to a solution of trimethyl sulfoxonium iodide (561 mg, 165 mmol, 2.5 equiv.) in MeCN (10 mL). The resulting mixture was stirred for 30 minutes at room temperature. To this solution was added the previously formed azine in MeCN (5 mL) and the reaction mixture was stirred at room temperature for 1h. The crude material was then filtered, concentrated *in vacuo* and purified by column chromatography (hexane:EtOAc = 95:1) to give the desired product N-iminyl aziridine (283 mg, 93%) as a slightly yellow oil.

^{1}H-NMR (600 MHz, $CDCl_3$) δ 7.60-7.56 (m, 2H), 7.40-7.31 (m, 6H), 7.25-7.24 (m, 2H), 7.21-7.16 (m, 3H), 7.00-6.98 (m, 2H), 3.04-2.99 (dd, *J* = 7.7, 4.7 Hz, 1H), 2.55 (d, *J* = 7.7 Hz, 1H), 2.26 (d, *J* = 4.7 Hz, 1H).

^{13}C-NMR (150 MHz, $CDCl_3$) δ 167.0, 138.5, 138.3, 135.8, 129.9, 129.3, 128.6, 128.29, 128.26, 128.1, 128.0, 127.0, 126.3, 45.9, 42.6.

HRMS (ESI) calcd. for $C_{21}H_{19}N_2$ $[M+H]^+$: 299.1548, found: 299.1545.

IR (neat) ν max cm^{-1}: 3058, 3029, 2986, 2926, 1660, 1603, 1561, 1493, 1444, 768, 746, 693, 673.

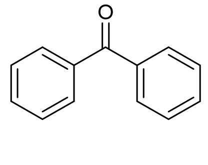

Benzophenone:

In a pressure vial aziridine **9** (1 equiv.) is diluted in DMSO ([0.5 M]) and immediately heated in an oil bath at 150 °C. After 24 hours the mixture is cooled to room temperature and the reaction then diluted with a saturated aqueous solution of NH_4Cl and extracted with EtOAc (3x). The combined organic layers were washed with brine, dried over anhydrous $MgSO_4$ and concentrated under reduced pressure. The crude product was purified by flash column chromatography on silica gel to afford the desired product. All spectroscopic data collected on the so obtained compound perfectly matched a sample of benzophenone obtained from Sigma-Aldrich.

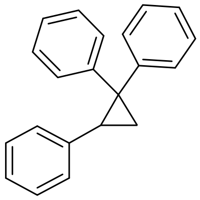

1,1,2-Triphenylcyclopropane (10):

To a solution of aziridine **7** (50 mg, 0.168 mmol, 1.0 equiv.) in toluene (2 ml) was added $Rh(OAc)_2$ (7.45 mg, 0.017 mmol, 0.01 equiv.) at room temperature and the reaction temperature was heated to 90 °C. The conversion was followed by TLC, the solvent was removed and the crude mixture was filtrated on celite® and purified by column chromatography (hexane:EtOAc = 4:1 ratio). The product was obtained in 41 mg (90 % yield). Data in accordance with the literature.[30]

^{1}H-NMR (400 MHz, $CDCl_3$) δ 7.35-7.27 (m, 6H), 7.19-7.05 (m, 7H), 6.87-6.86 (m, 2H), 2.85 (dd, J = 8.9, 6.5 Hz, 1H), 1.98 (dd, J = 6.5, 5.3 Hz, 1H), 1.80 (dd, J = 8.9, 5.3 Hz, 1H).

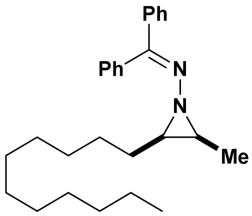

***N*-(*cis*-2-Methyl-3-undecylaziridin-1-yl)-1,1-diphenylmethanimine (*cis*-11):**

The title compound was isolated after preparative TLC.

^{1}H-NMR (600 MHz, $CDCl_3$) δ 7.53-7.28 (m, 10H), 2.10-1.98 (m, 2H), 1.29-1.25 (m, 20H), 0.88 (t, *J* = 6.9 Hz, 3H), 0.86-0.84 (bd, 3H).

^{13}C-NMR (150 MHz, $CDCl_3$) δ 129.34, 129.01 (2C), 128.41, 128.19 (2C), 127.78 (2C), 127.65 (2C), 47.02, 44.57, 32.07, 29.85, 29.79, 29.72, 29.68, 29.63, 29.59, 29.51, 28.08, 22.85, 14.29, 12.08. [n.b. 3 quaternary carbon are not listed].

HRMS (ESI) calcd. for $C_{27}H_{39}N_2$ $[M+H]^+$: 391.3108, found: 391.3105.

IR (neat) ν max cm^{-1}: 3058, 3025, 2921, 2852, 1492, 1462, 1444, 1383, 1318, 1298, 774, 693.

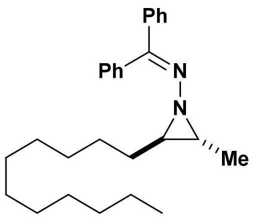

***N*-(*trans*-2-Methyl-3-undecylaziridin-1-yl)-1,1-diphenylmethanimine (*trans*-11):**

The title compound was isolated after preparative TLC.

^{1}H-NMR (600 MHz, $CDCl_3$) δ 7.51-7.28 (m, 10H), 2.06-2.01 (bm, 2H), 1.31-1.23 (bm, 20H), 0.88 (t, *J* = 6.5 Hz, 3H), 0.79 (d, *J* = 5.0 Hz, 3H).

^{13}C-NMR (150 MHz, $CDCl_3$) δ 164.58, 138.33, 136.85, 129.47, 129.00 (2C), 128.44, 128.37 (2C), 128.18 (2C), 127.76 (2C), 48.95, 44.55, 32.07, 29.81, 29.79, 29.72, 29.68, 29.58, 29.50, 27.31, 26.98, 22.84, 14.29, 12.08.

HRMS (ESI) calcd. for $C_{27}H_{39}N_2$ $[M+H]^+$: 391.3108, found: 391.3105.

IR (neat) ν max cm^{-1}: 3058, 3025, 2921, 2852, 1587, 1560, 1492, 1462, 1444, 1441, 1383, 1318, 1298, 774, 721, 693, 670, 649.

Supplementary Figures

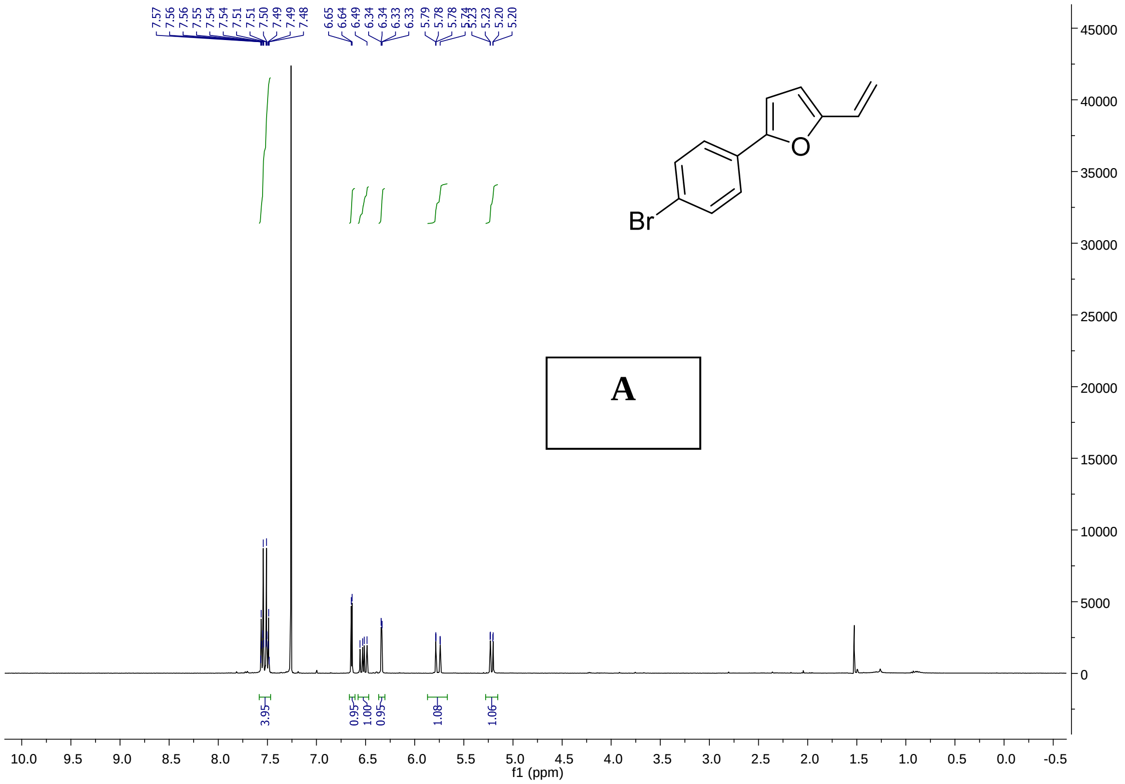

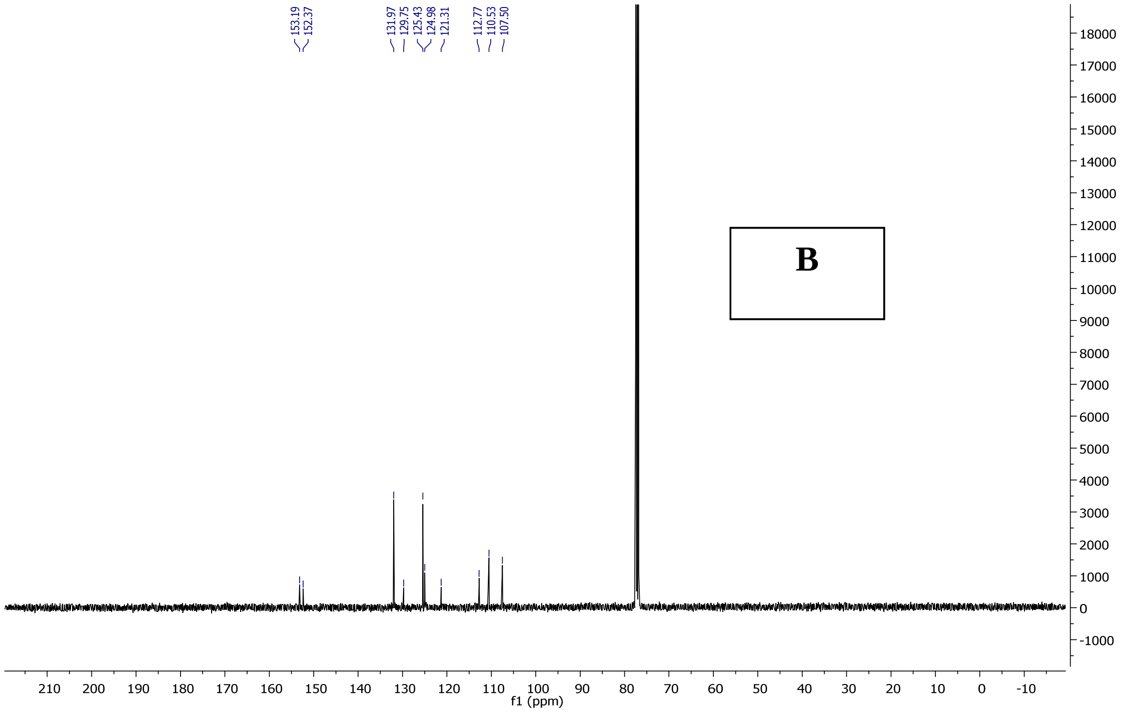

Supplementary Figure 1. (**A**) ^{1}H NMR and (**B**) ^{13}C NMR spectra of 2-(4-Bromophenyl)-4-vinylfuran (**3l**)

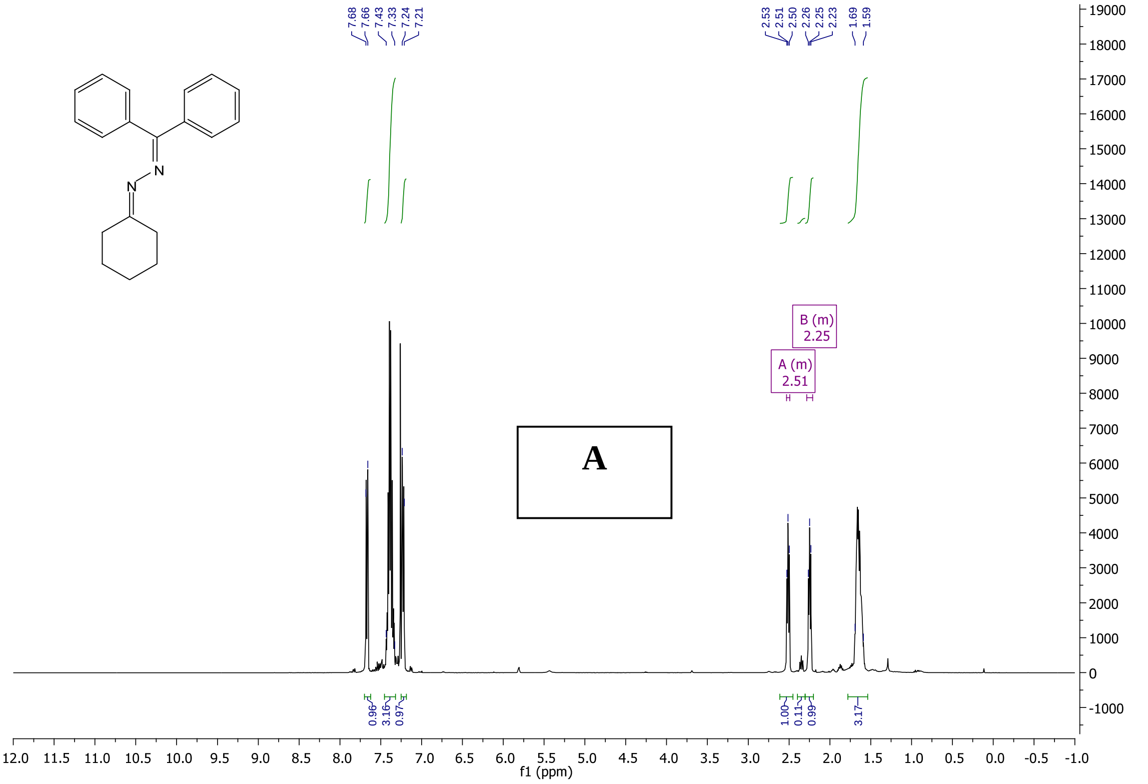

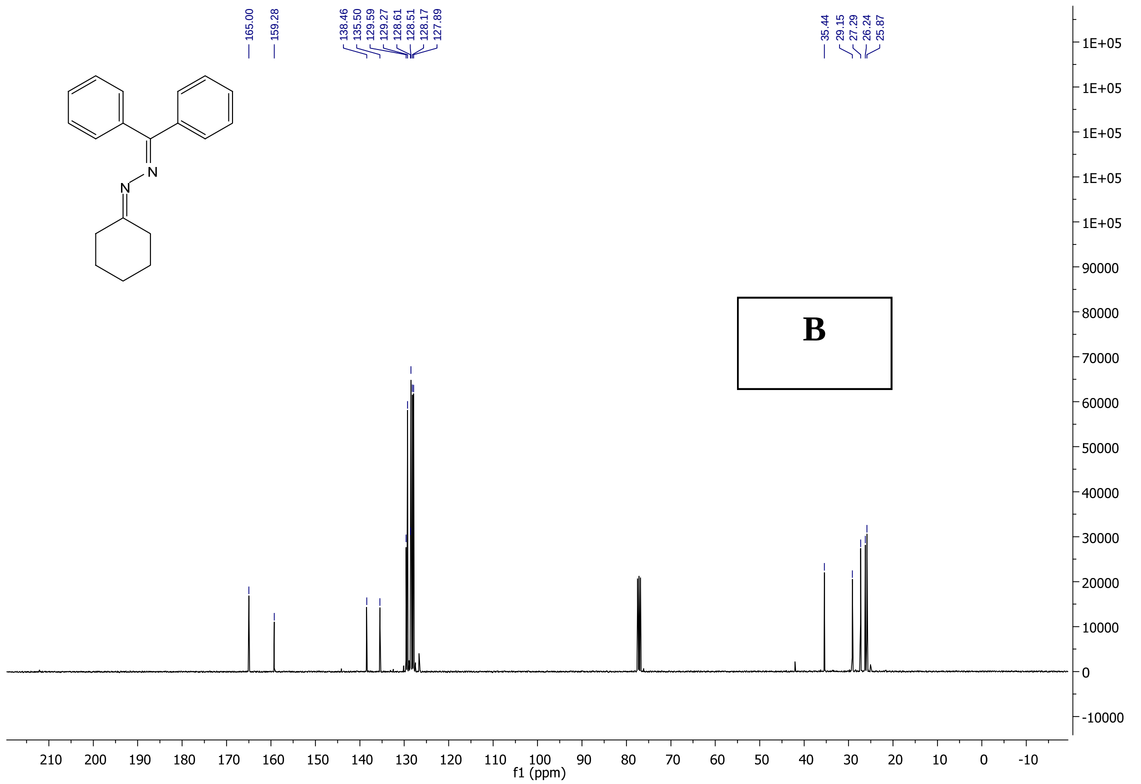

Supplementary Figure 2. (**A**) ^{1}H NMR and (**B**) ^{13}C NMR spectra of 1-Cyclohexylidene-2-(diphenylmethylene)hydrazine (**SI-1**).

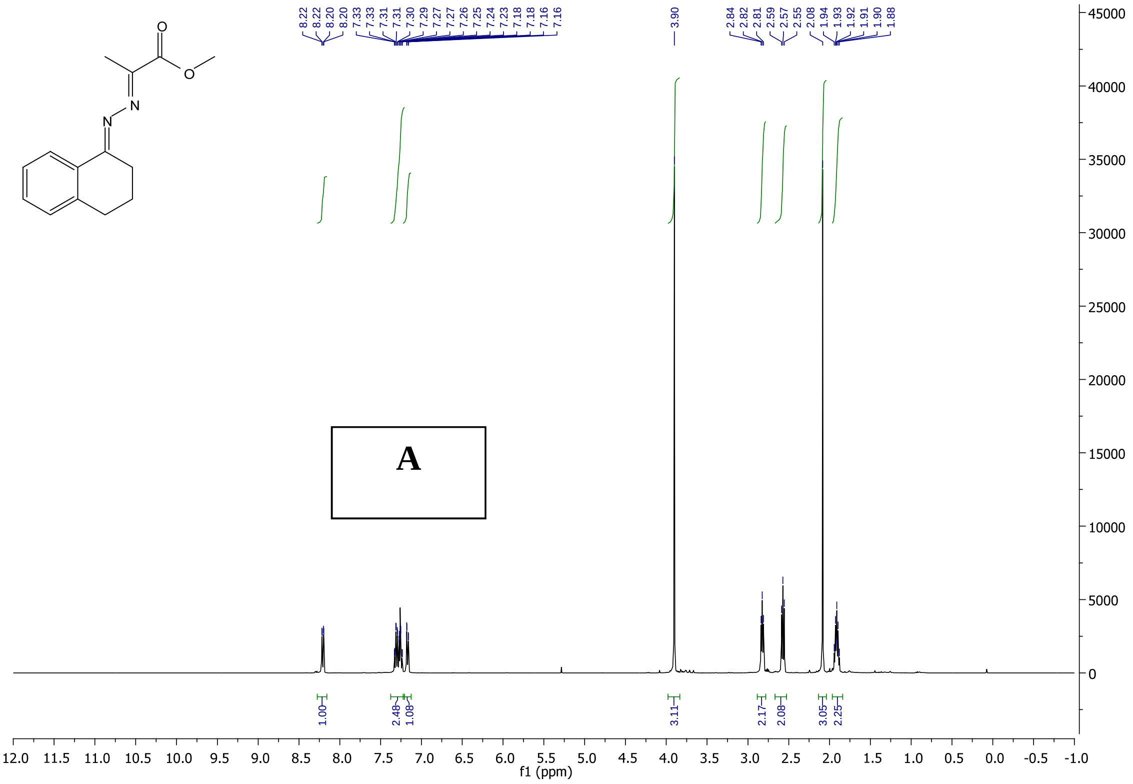

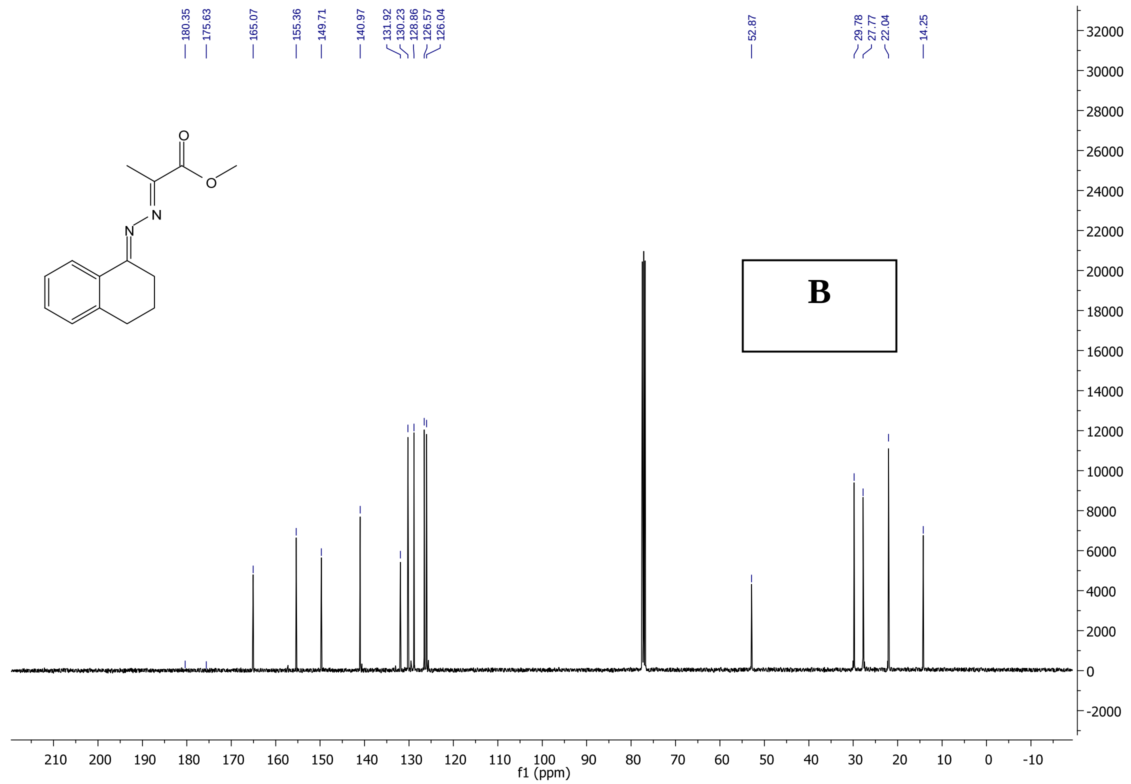

Supplementary Figure 3. (**A**) ^{1}H NMR and (**B**) ^{13}C NMR spectra of Methyl (*E*)-2-(((*E*)-3,4-dihydronaphthalen-1(2*H*)-ylidene)hydrazono)propanoate (**SI-2**)

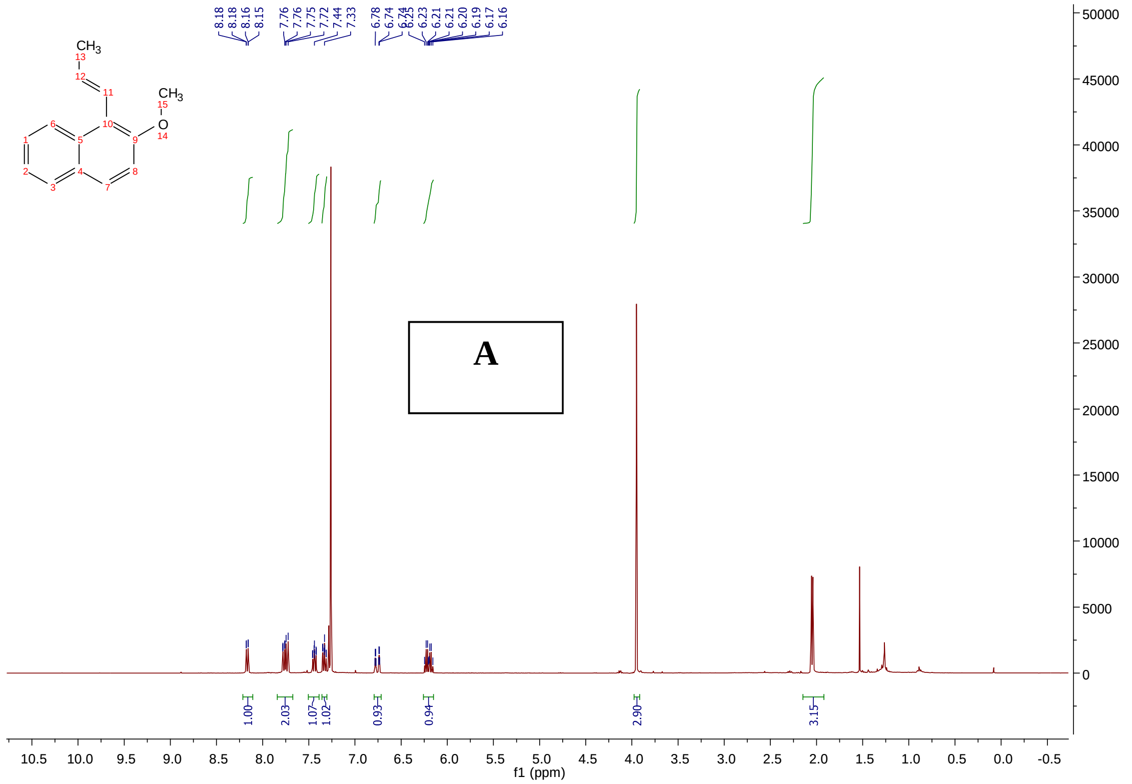

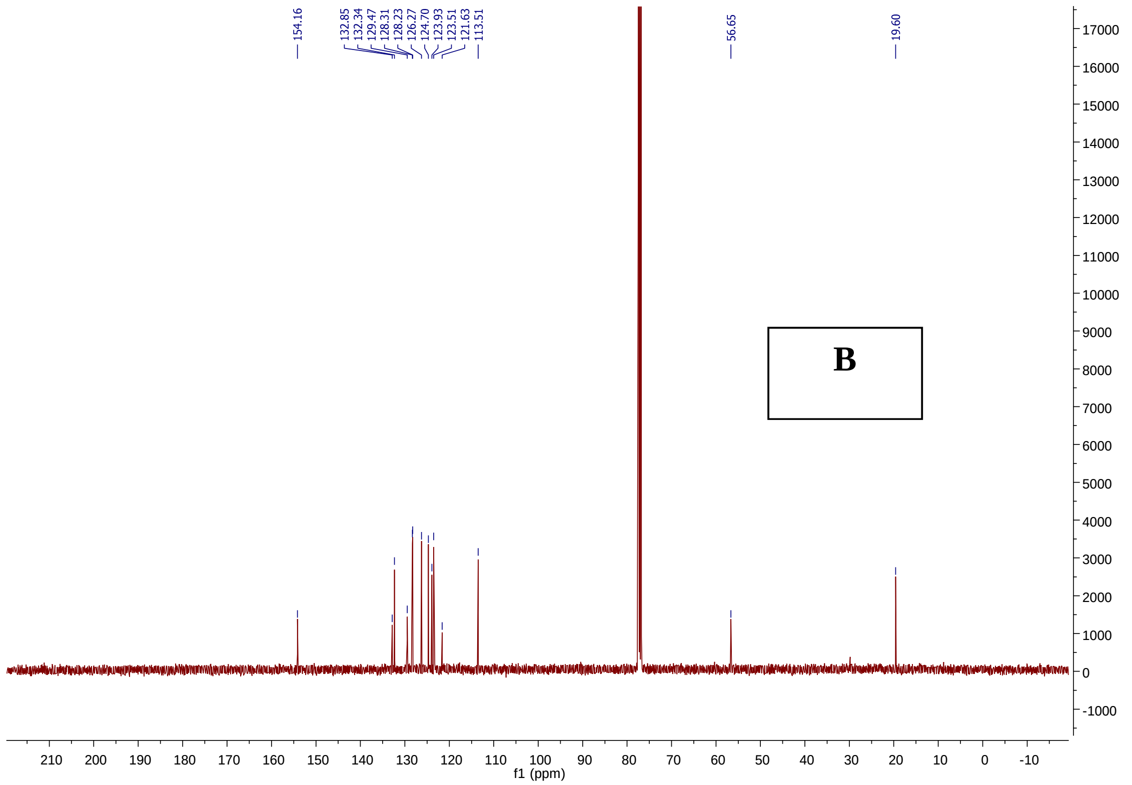

Supplementary Figure 4. (**A**) ^{1}H NMR and (**B**) ^{13}C NMR spectra of (*E*)-2-Methoxy-1-(prop-1-en-1-yl)naphthalene (**7b**)

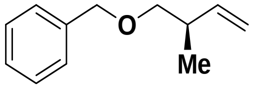

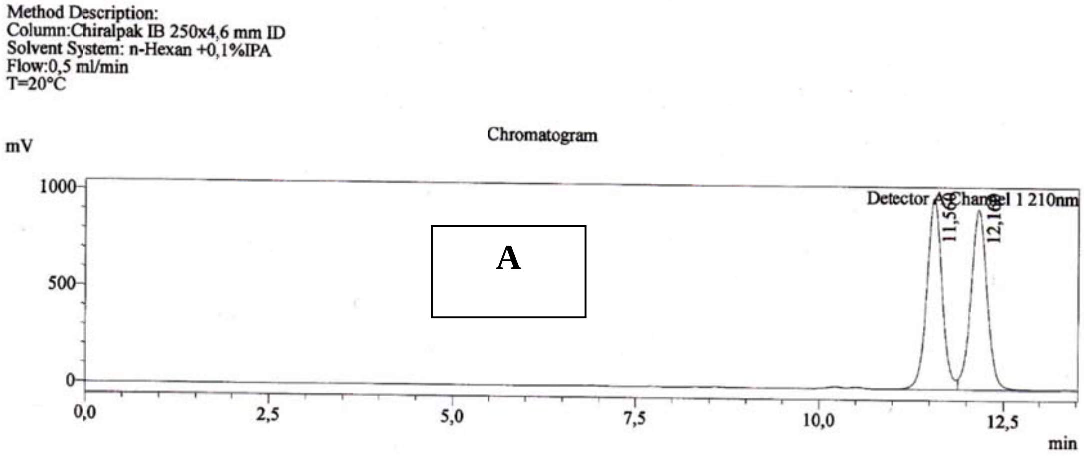

Detector A Channel 1 210nm

Peak#	Ret. Time	Area	Area%
1	11,560	14265703	50,074
2	12,160	14223645	49,926
Total		28489348	100,000

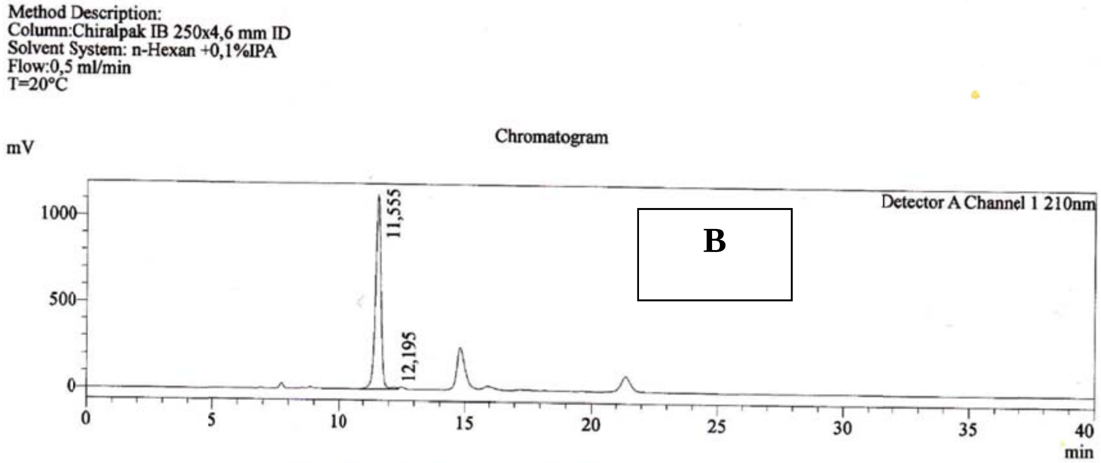

Detector A Channel 1 210nm

Peak#	Ret. Time	Area	Area%
1	11,555	16970363	98,911
2	12,195	186822	1,089
Total		17157185	100,000

Supplementary Figure 5. HPLC traces of (**A**) racemic and (**B**) enantioenriched (((2-Methylbut-3-en-1-yl)oxy)methyl)benzene (**8**)

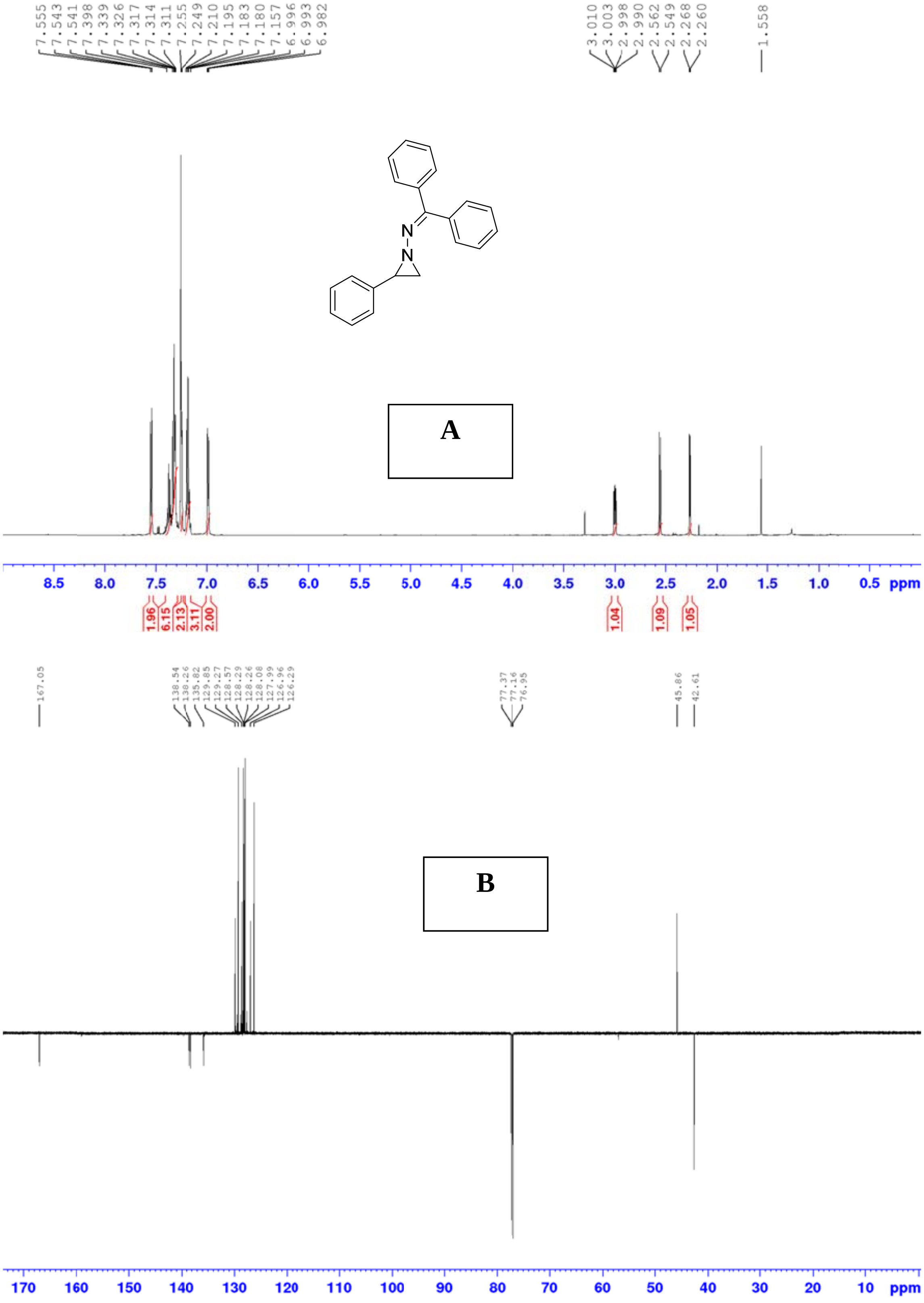

Supplementary Figure 6. (**A**) ^{1}H NMR and (**B**) ^{13}C NMR spectra of 1,1-Diphenyl-*N*-(2-phenylaziridin-1-yl)methanimine (**9**)

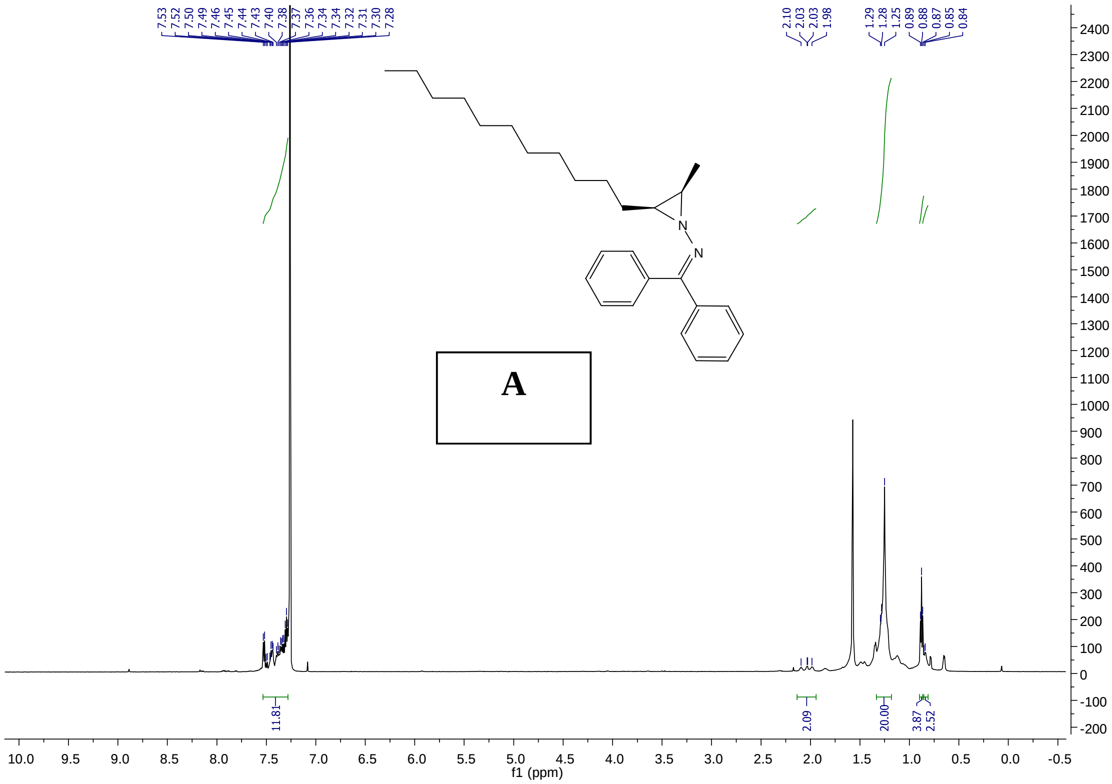

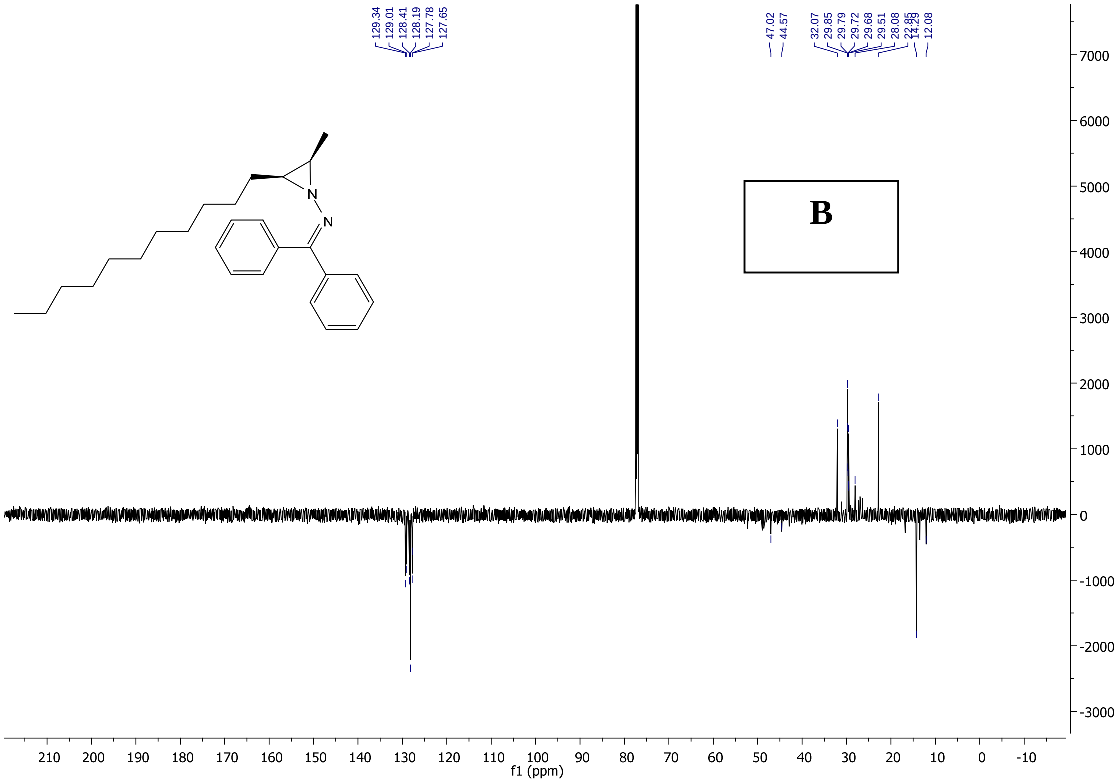

Supplementary Figure7. (**A**) ^{1}H NMR and (**B**) ^{13}C NMR spectra of *N*-(*cis*-2-Methyl-3-undecylaziridin-1-yl)-1,1-diphenylmethanimine (*cis*-**11**)

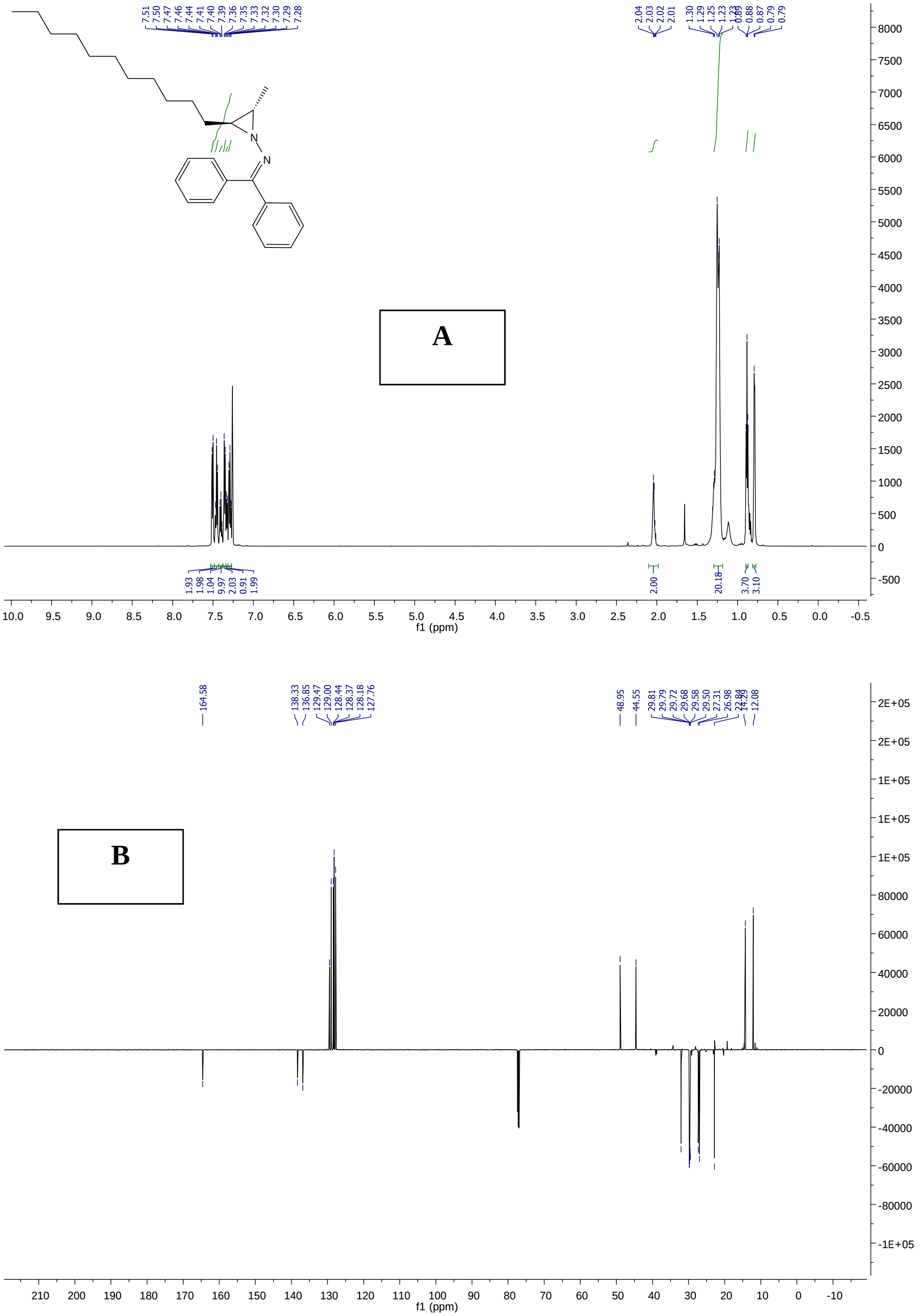

Supplementary Figure 8. (**A**) ^{1}H NMR and (**B**) ^{13}C NMR spectra of *N*-(*trans*-2-Methyl-3-undecylaziridin-1-yl)-1,1-diphenylmethanimine (*trans*-**11**)

Supplementary references

1. Zhao, R. & Shea, K.J. *ACS Macro Lett.*, **4**, 584-587 (2015).

2. Edwards, M.G., Paxton, R.J., Pugh, D.S., Whitwood, A.C. & Taylor, R.J.K., *Synthesis*, **20**, 3279-3288 (2008).

3. Bagwell, C.L., Leonard, D.M.L., Griffiths, J-P., Moloney, M.G., Stratton, N.J. & Travers, D.P. *Macromol. React. Eng.*, **8**, 170-180 (2014).

4. Hu, M., Ni, C., Li, L., Han, Y. & Hu, J. *J. Am. Chem. Soc.*, **45**, 14496-1451 (2015).

5. Zhu, C.,Bi, B., Yao, D., Chen, Q-Y. & Luo, Z-B. *Synthesis*, **47**, 1877–1886 (2015).

6. Schmitt, E., Landelle, G., Vors, J-P., Lui, N., Pazenok, S. & Leroux, F.R. *Eur. J. Org. Chem.*, 6052-6060 (2015).

7. Gøgsig, T. M., Søbjerg, L. S., Lindhardt, A. T., Jensen, K. L. & Skrydstrup, T., *J. Org. Chem*., **73**, 3404–3410 (2008).

8. A. L. L. Hansen, A. Murray, D. Tanner, *Org. Biomol. Chem.* **2006**, *4*, 4497–4505.
9. Ikawa, T., Sajiki, H. & Hirota, K., *Tetrahedron*, **61**, 2217–2231 (2005).
10. Li, J. H., Tang, B. X., Tao, L. M., Xie, Y. X., Liang, Y. & Zhang, M. B., *J. Org. Chem.*, **71**, 7488−7490 (2006).

11. W. Ren, M. Yamane, *J. Org. Chem.* **2006**, *74*, 8332−8335.Zhao, R. & Shea, K.J. *ACS Macro Lett.*, **4**, 584-587 (2015).

12. Kawashima, S., Aikawa, K. & Mikami, K., *Eur. J. Org. Chem*., 3166–3170 (2016).
13. Yu, J-Y. & Kuwan, R., *Angew. Chem. Int. Ed.*, **48**, 7217–7220 (2009).

14. Bouraiou, A., Debache, A., Rhouati, S., Carboni, B. & Belfaitah, A. *J. Heterocyclic Chem.*, **45**, 329–333 (2008).

15. Waser, J., Gaspar, B., Nambu, H. & Carreira, E. M., *J. Am. Chem. Soc.*, **128**, 11693–11712 (2006).

16. Deb, A., Manna, S., Modak, A., Patra, T., Maity, S. & Maiti, D. *Angew. Chem. Int. Ed.*, **52**, 9747–9750 (2013).

17. Mundal, D. A., Lutz, K. E. & Thomson, R. J., *Org. Lett.*, **11**, 465–469 (2009).

18. Chen, J., & Che, C-M, *Angew. Chem. Int. Ed.*, **43**, 4950–4954 (2004).

19. Khan, F.A. &. Budanur, B.M, *Tetrahedron.* B.M, **71**, 7600–7604 (2015).

20. Watkins, A. L. & Landis, C, R., *Org. Lett.*, **13**, 164–167 (2011).

21. Abramovitch, A. & Marek, I., *Eur. J. Org. Chem.,* 4924–4931 (2008).

22. Coombs, J. R., Haeffner, F., Kliman, L. T. & Morken, J. P., *J. Am. Chem. Soc.*, **135**, 11222–11231 (2013).

23. Sinha, S. C. , Sinha-Bagchi, A. & Keinan, E. *J. Org. Chem.*, **58**, 7789–7796 (1993).

24. Chow, S. & Kitching, W. *Tetrahedron: Asymmetry*, **13**, 779–793 (2002).

25. Kumar, R. , Sharma, A., Sharma, N., Kumar, V. & Sinha, A. K. *Eur. J. Org. Chem*., 5577–5582 (2008).

26. Wu, S-W., Liu, J-L.& Liu, F. *Org. Lett.*, **18**, 1–3 (2016).

27. Feldhues, M. & Schäfer, H.J. *Tetrahedron*, **41**, 4213-4235 (1985).

28. Calo, V., Lopez, L. & Carlucci, W. *J. Chem. Soc., Perkin Trans. I*, **1983**, 2953-2956 (1983).

29. Lebel, H. & Paquet, V., *J. Am. Chem. Soc.*, **126**, 320–328 (2004).

30. Li, Y., Huang, J.-S., Zhou, Z.-Y. & Che, C-M. *J. Am. Chem. Soc.*, **123**, 4843–4844 (2001).
